# Supplementary material for: Neuronal birthdate reveals topography in a vestibular brainstem circuit for gaze stabilization
Source: Curr Biol. Author manuscript; Available in PMC 2023 Apr 12. (PMC10089979; doi:10.1016/j.cub.2023.02.048)
Supplement: MMC1 [file NIHMS1877847-supplement-MMC1.pdf]

Current Biology, Volume *33*

Supplemental Information

Neuronal birthdate reveals topography in a vestibular brainstem circuit for gaze stabilization

Dena Goldblatt, Stephanie Huang, Marie R. Greaney, Kyla R. Hamling, Venkatakaushik Voleti, Citlali Perez-Campos, Kripa B. Patel, Wenze Li, Elizabeth M.C. Hillman, Martha W. Bagnall, and David Schoppik

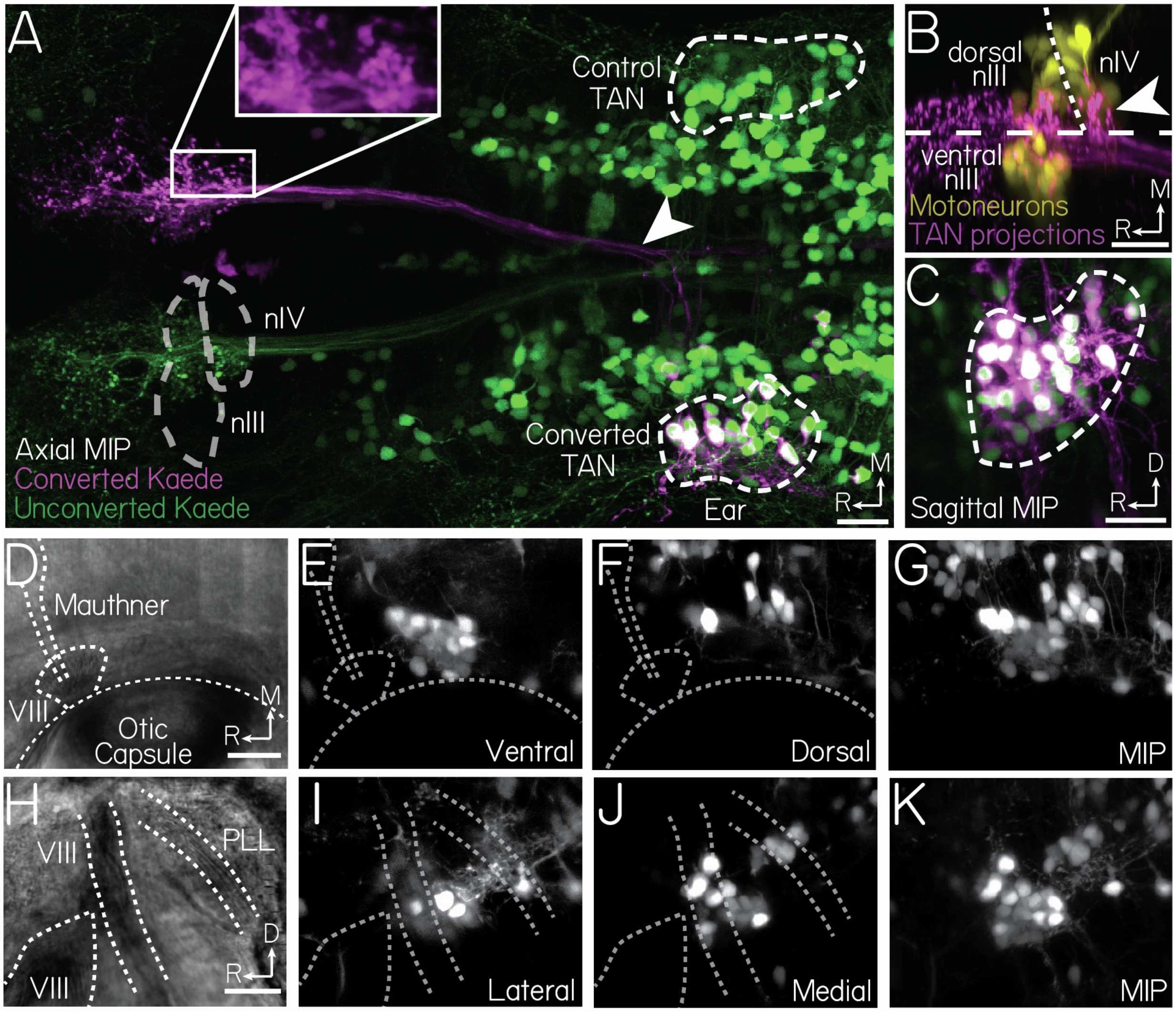
A
Control TAN
nIV
nIII
Axial MIP
Converted Kaede
Unconverted Kaede
Converted TAN
Ear
M
R
B
dorsal nIII
nIV
ventral nIII
Motoneurons
TAN projections
M
R
C
Sagittal MIP
D
R
D
Mauthner
VIII
Otic Capsule
M
R
E
Ventral
F
Dorsal
G
MIP
H
VIII
PLL
VIII
D
R
I
Lateral
J
Medial
K
MIP

Figure S1: Localization of the tangential vestibular nucleus, Related to Figure 1. (A) Retrograde photofill technique. Maximum intensity projection (MIP) of the tangential vestibular nucleus and its ascending projections at 5 dpf. White box shows conversion target region. White arrow points to ascending projections. Grey dashed lines outline the position of the extraocular motor nuclei. White dashed lines outline the bounds of photofilled soma in the tangential vestibular nucleus. **(B)** Photoconversion target region. White arrow points to the characteristic axonal arborizations at cranial nucleus IV (nIV). Motor neurons labeled in yellow. Ascending projections labeled in magenta. Horizontal dashed line indicates midline. Diagonal white dashed line shows midbrain-hindbrain boundary. **(C)** Center-surround appearance of converted (magenta) and unconverted (green) Kaede in retrogradely-labeled soma. (D-G) Location of the tangential vestibular nucleus in an axial view. **(D)** Transmitted light view of the tangential vestibular nucleus, 15 µm ventral to the base. Grey dashed lines outline the otic capsule, VIII$_{th}$ cranial nerve, and lateral dendrite of the Mauthner neuron. **(E)** Ventral plane, approximately 15 µm dorsal to the base. Landmarks in D labeled as grey dashed lines. **(F)** Dorsal plane, approximately 35 µm dorsal to the base. **(G)** Maximum intensity projection. (H-K) Location of the tangential vestibular nucleus in a sagittal view. **(H)** Transmitted light view, 10 µm from the lateral edge. White dashed lines outline the entry point and ascending branch of the VIII$_{th}$ nerve (VIII) and the posterior lateral line branch (PLL). **(I)** Lateral plane, approximately 10 µm medial to the lateral edge. **(J)** Medial plane, approximately 30 µm medial to the lateral edge. **(K)** Maximum intensity projection. All scale bars 20 µm.

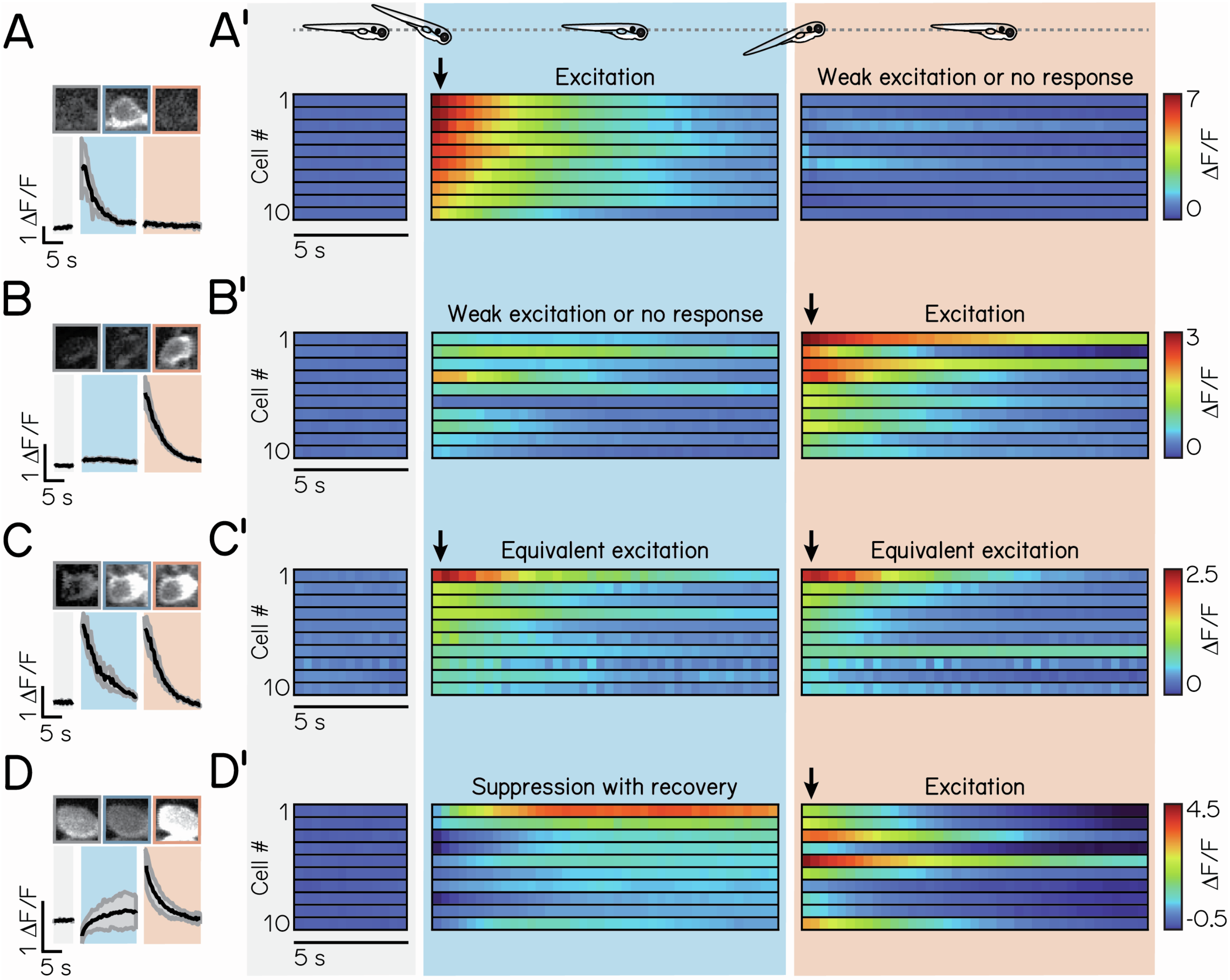
A
A'
B
B'
C
C'
D
D'
Excitation
Weak excitation or no response
Weak excitation or no response
Excitation
Equivalent excitation
Equivalent excitation
Suppression with recovery
Excitation
Cell #
1
10
5 s
1 ΔF/F
ΔF/F
7
0
3
0
2.5
0
4.5
-0.5

Figure S2: Response profiles of tangential vestibular neurons to tonic pitch-tilt rotations, related to Figure 2. (A) Example response from a nose-down neuron with no activity to the non-preferred direction. Black line indicates mean response across three stimulus repeats; shaded bars show standard deviation. Same example neuron as in Fig. 2D. **(A')** Heatmap showing the timecourse of calcium activity for n=10 example nose-down neurons with no response or weak excitation to the non-preferred (nose-up) direction. Each row represents a distinct neuron. Shaded bars indicate time at horizontal for baseline (grey), following the nose-down rotation (blue), and following the nose-up rotation (orange). Black vertical arrow points to excitatory responses used to assign a nose-up, nose-down, or no selectivity identity. **(B)** Example response from a nose-up neuron with no activity to the non-preferred direction. Same example neuron as in Fig. 2D. **(B')** Heatmap showing the timecourse of calcium activity for n=10 example nose-up neurons with no response or weak excitation to the non-preferred (nose-down) direction. **(C)** Example response from neuron with no directional selectivity. Same example neuron as in Fig. 2D. **(C')** Heatmap showing the timecourse of calcium activity for n=10 example neurons with no directional selectivity. **(D)** Example response from nose-up neuron that exhibits suppression with recovery to the non-preferred (nose-down) direction. **(D')** Heatmap showing the timecourse of calcium activity for n=10 example nose-up neurons that exhibit suppression with recovery to the non-preferred direction.

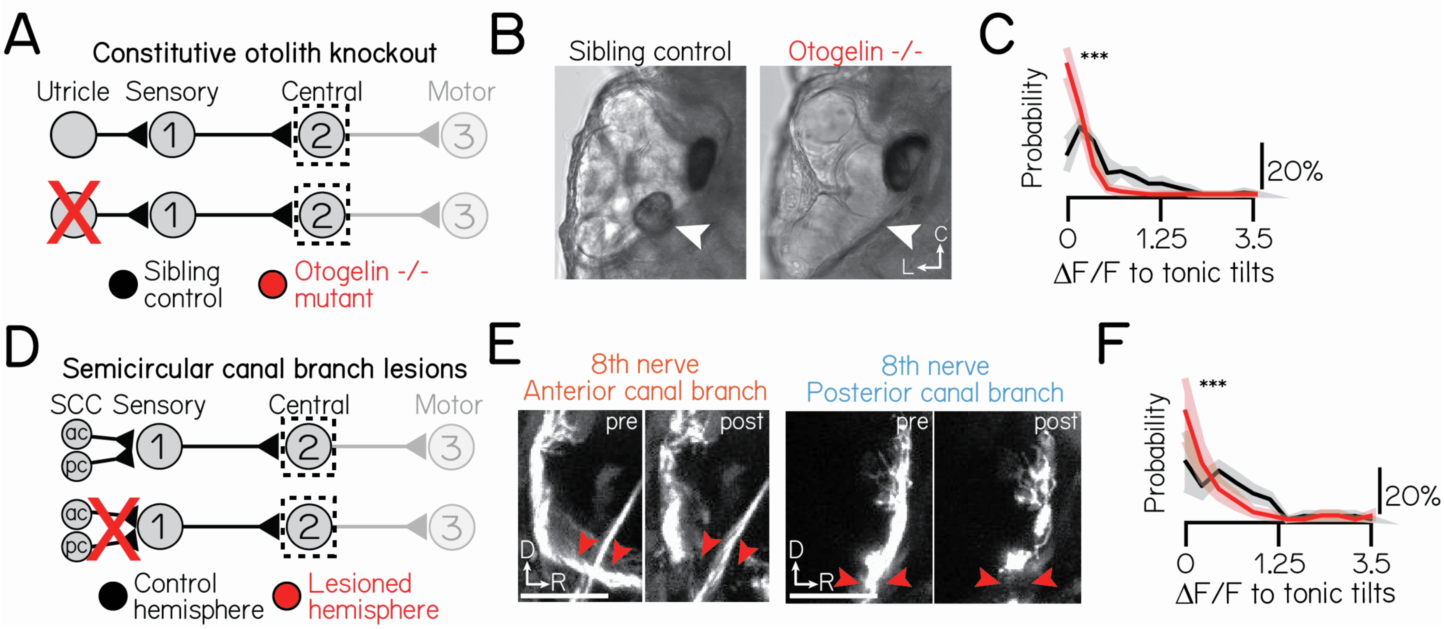
A
Constitutive otolith knockout
Utricle
Sensory
Central
Motor
Sibling control
Otogelin -/- mutant
B
Sibling control
Otogelin -/-
C
L
C
Probability
***
20%
0
1.25
3.5
ΔF/F to tonic tilts
D
Semicircular canal branch lesions
SCC Sensory
Central
Motor
ac
pc
Control hemisphere
Lesioned hemisphere
E
8th nerve
Anterior canal branch
8th nerve
Posterior canal branch
pre
post
D
R
F
Probability
***
20%
0
1.25
3.5
ΔF/F to tonic tilts

Figure S3: Responses to tonic pitch-tilt rotations originate primarily from the utricular otoliths, Related to Figure 2. (A) Circuit schematic for utricular otolith knockout experiments. Black dashed lines outline the tangential nucleus as the circuit element of focus. **(B)** Example images from a control larvae (left) and an otogelin null mutant (right). White arrow points to the position of the utricular otolith. **(C)** Probability distributions of the maximum ΔF/F response to preferred directional pitch-tilts in control (black) and otogelin mutants (red). Solid lines shows mean from jackknife resampling; shaded bars, standard deviation. Controls: n=144 neurons, N=3 fish; Mutants: n=183 neurons, N=3 fish. **(D)** Circuit schematic for uni-lateral VIII_{th} nerve lesions (anterior and posterior semicircular canal branches). **(E)** Example images from larvae before and after uni-lateral VIII_{th} nerve lesions. Left and right image sets show the anterior and posterior semicircular canal branches, respectively, before and after lesion. Both branches are lesioned in each experiment. Red arrows point to lesion sites. **(F)** Probability distributions of the maximum ΔF/F response to preferred directional pitch-tilts in control (black) and lesioned (red) hemispheres. Solid lines shows mean from jackknife resampling; shaded bars, standard deviation. Control hemisphere: n=57 neurons, N=4 fish; Lesioned hemisphere: n=49 neurons, N=4 fish. All scale bars, 20 µm. All data: Three stars denotes significance at the $p<0.001$ level.

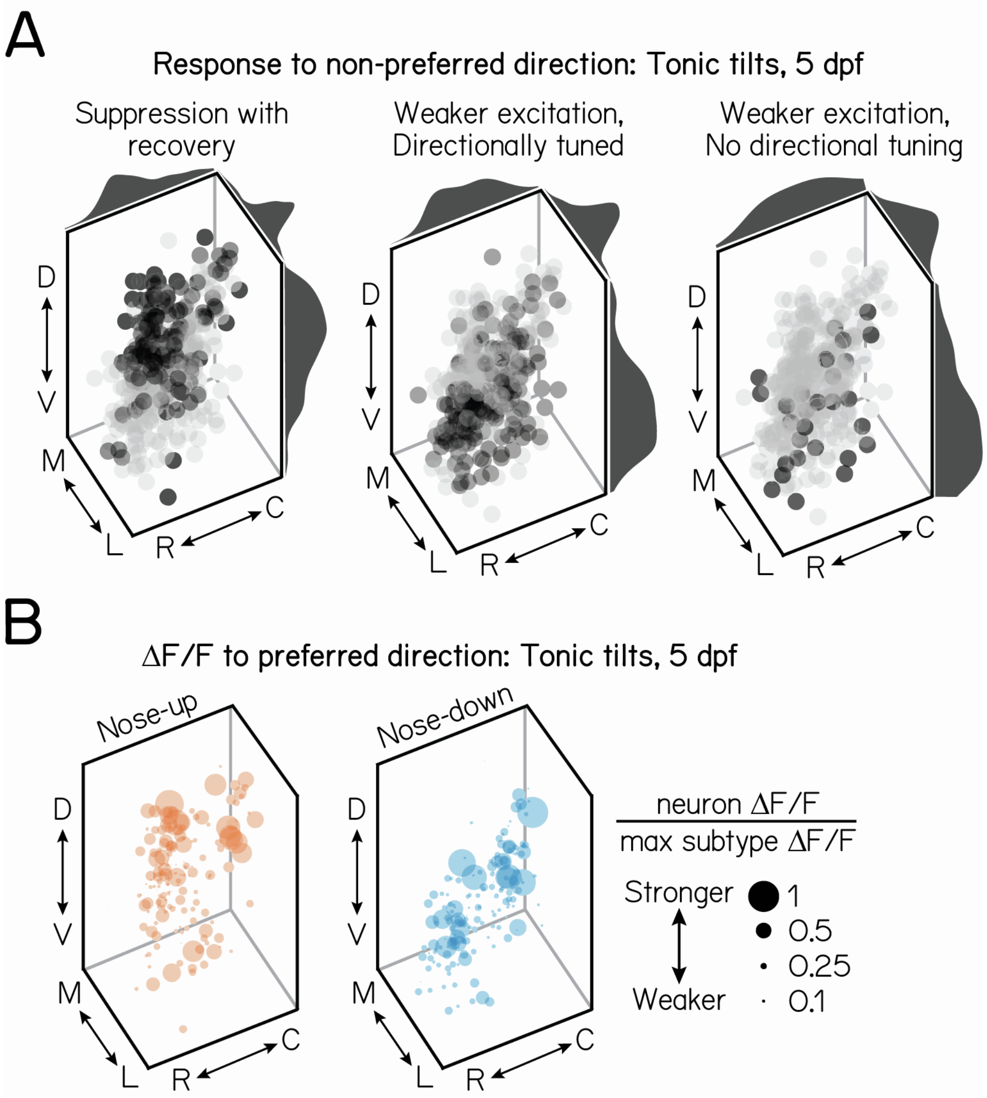

A
Response to non-preferred direction: Tonic tilts, 5 dpf
Suppression with recovery
Weaker excitation, Directionally tuned
Weaker excitation, No directional tuning
D
V
M
L
R
C
B
ΔF/F to preferred direction: Tonic tilts, 5 dpf
Nose-up
Nose-down
neuron ΔF/F
max subtype ΔF/F
Stronger
Weaker
1
0.5
0.25
0.1

Figure S4: Projection neuron soma are locally organized according to similarities in tilt responses, Related to Figure 3. (A) Soma position of projection neurons with each response type to their non-preferred, tonic tilt direction. Dark grey shows neurons with the given response; light grey shows all other neurons. The directional tuning of neurons with weak excitation to non-preferred directions is determined by their directional tuning index (>0.1 or ≤0.1). Marginal distributions illustrate the probability of soma position in each spatial axis. Data from the same neurons shown in Figure 3G. **(B)** Soma position of nose-up (left) and nose-down (right) projection neurons, scaled according to the strength of their calcium response (ΔF/F) to tilts relative to the strongest response observed for that subtype. Larger circles indicate stronger responses. Data from the same neurons shown in Figure 3G.

Afferent source	1	2	3	4	5	6	7	8	9	10	11	12	13	14	15	16	17	18	19	Tuning
Ear_AnteriorCrista_R_01	0	0	0	0	0	0	0	0	0	0	0	0	8	0	0	0	0	0	0	
Ear_AnteriorCrista_R_02	0	0	0	0	**2**	0	0	0	0	0	0	0	0	**8**	0	0	0	0	0	
Ear_AnteriorCrista_R_03	0	0	0	0	0	0	0	0	**3**	0	0	0	0	0	0	0	0	0	0	
Ear_AnteriorCrista_R_04	0	0	0	0	**1**	0	0	0	0	0	0	**9**	0	0	0	0	0	0	0	
Ear_AnteriorCrista_R_05	0	0	0	0	0	0	0	0	**2**	0	0	0	0	0	0	0	0	0	**12**	
Ear_AnteriorCrista_R_06	0	0	0	0	0	0	0	0	**5**	0	0	0	0	0	0	0	0	0	0	
Ear_PosteriorCrista_R_01	0	0	0	0	0	0	0	0	0	0	0	0	0	0	0	0	0	0	0	
Ear_PosteriorCrista_R_02	0	0	0	0	0	**2**	0	0	0	**2**	0	0	0	0	0	0	**8**	0	0	
Ear_PosteriorCrista_R_03	0	0	0	0	0	0	0	0	0	0	0	0	0	0	0	0	0	7	0	
Ear_PosteriorCrista_R_04	0	0	0	0	0	**3**	0	0	0	**1**	0	0	0	0	**10**	0	0	0	0	
Ear_PosteriorCrista_R_05	0	0	0	0	0	0	0	0	0	**1**	0	0	0	0	0	**5**	0	0	0	
Ear_AnteriorMacula_R_01	**1**	0	**2**	**3**	0	0	0	0	0	0	0	0	0	0	0	0	0	0	0	6.00894
Ear_AnteriorMacula_R_02	0	**3**	0	0	0	0	0	0	0	0	0	0	0	0	0	0	0	0	0	6.09989
Ear_AnteriorMacula_R_03	**1**	0	0	0	0	0	0	0	0	0	0	0	0	0	0	0	0	0	0	6.08107
Ear_AnteriorMacula_R_04	0	**3**	**1**	0	0	**1**	0	0	0	0	0	0	0	0	0	0	0	0	0	5.92756
Ear_AnteriorMacula_R_05	0	0	0	0	0	0	**1**	0	0	0	0	0	0	0	0	0	0	0	0	0.204992
Ear_AnteriorMacula_R_06	**1**	0	0	0	**1**	0	**1**	0	0	0	0	0	0	0	0	0	0	0	0	0.523691
Ear_AnteriorMacula_R_07	0	0	0	**1**	0	0	0	0	0	0	0	0	0	0	0	0	0	0	0	0.825107
Ear_AnteriorMacula_R_08	0	0	0	0	**1**	0	0	0	0	0	**1**	0	0	0	0	0	0	0	0	1.37515
Ear_AnteriorMacula_R_09	**1**	0	**4**	0	0	0	0	0	0	0	0	0	0	0	0	0	0	0	0	6.04532
Ear_AnteriorMacula_R_10	0	0	0	0	0	0	0	0	0	0	0	0	0	0	0	0	0	0	0	1.21203
Ear_AnteriorMacula_R_11	0	0	0	0	0	0	0	0	0	0	0	0	0	0	0	0	0	0	0	4.20026
Ear_AnteriorMacula_R_12	0	0	0	**2**	0	0	0	**2**	0	0	0	0	0	0	0	0	0	0	0	1.61526
Ear_AnteriorMacula_R_13	0	0	0	0	0	0	**1**	0	0	0	0	0	0	0	0	0	0	0	0	0.257178
Ear_AnteriorMacula_R_14	0	0	0	0	0	0	0	0	0	0	0	0	0	0	0	0	0	0	0	3.09177
Ear_AnteriorMacula_R_15	0	0	0	0	0	0	0	**2**	0	0	0	0	0	0	0	0	0	0	0	0.93221
Ear_AnteriorMacula_R_16	0	0	0	0	0	0	0	0	0	0	0	0	0	0	0	0	0	0	0	4.19152
Ear_AnteriorMacula_R_17	0	0	0	0	0	0	**1**	0	0	0	0	0	0	0	0	0	0	0	0	1.31298

Table 1: Number of synapses between 28 VIIIth nerve afferents (crista: semicircular canals; macula: utricular otoliths) and 19 projection neurons, Related to Figure 5. “Tuning” is an estimate of the sensitivity derived from the orientation of the macular hair cells, as described in[55]; values between 0 and $\pi/2$ indicates rostral (nose-up) tuning. Projection neuron tuning (nose-up/nose-down) is defined as a weighted circular average of their utricular inputs. Projection neurons 1-3, 6, 11, 16-19 receive nose-up utricular input; neurons 6, 11, 16-19 also receive directionally-matched posterior cristae input. Projection neurons 4-5, 7-10, 12-15, 20 receive nose-down utricular input; neurons 5, 10, 13-15, and 20 also receive directionally-matched anterior cristae input.

Table S1: Number of synapses between 28 VIIIth nerve afferents (crista: semicircular canals; macula: utricular otoliths) and 19 projection neurons, Related to Figure 5. "Tuning" is an estimate of the sensitivity derived from the orientation of the macular hair cells, as described in[55]; values between 0 and $\pi/2$ indicates rostral (nose-up) tuning. Projection neuron tuning (nose-up/nose-down) is defined as a weighted circular average of their utricular inputs. Projection neurons 1-3, 6, 11, 16-19 receive nose-up utricular input; neurons 6, 11, 16-19 also receive directionally-matched posterior cristae input. Projection neurons 4-5, 7-10, 12-15, 20 receive nose-down utricular input; neurons 5, 10, 13-15, and 20 also receive directionally-matched anterior cristae input.
